# Femtosecond Spectroscopy of Au Hot-Electron Injection into TiO_2_: Evidence for Au/TiO_2_ Plasmon Photocatalysis by Bactericidal Au Ions and Related Phenomena

**DOI:** 10.3390/nano9020217

**Published:** 2019-02-06

**Authors:** Marina Radzig, Olga Koksharova, Inessa Khmel, Vladimir Ivanov, Khursand Yorov, John Kiwi, Sami Rtimi, Elina Tastekova, Arseny Aybush, Victor Nadtochenko

**Affiliations:** 1 Institute of Molecular Genetics, Russian Academy of Sciences, Kurchatov Sq. 2, 123182 Moscow, Russia; radzig@yandex.ru (M.R.); koksharova@genebee.msu.su (O.K.); khmel@img.ras.ru (I.K.); 2 A. N. Belozersky Institute of Physico Chemical Biology and Chemical Department of Lomonosov Moscow State University, 119992 Moscow, Russia; khursandy@gmail.com; 3 Kurnakov Institute of General and Inorganic Chemistry of the Russian Academy of Sciences, Leninskiy Av 31, 119991 Moscow, Russia; van@igic.ras.ru; 4 Ecole Polytechnique Fédérale de Lausanne, EPFL-SB-ISIC-GPAO, Station 6, CH-1015 Lausanne, Switzerland; john.kiwi@epfl.ch (J.K.); sami.rtimi@epfl.ch (S.R.); 5 N. N. Semenov Institute of Chemical Physics, Russian Academy of Sciences, Kosigin str. 4, 119991 Moscow, Russia; e.tastekova@fmlab.ru (E.T.); arseny.aybush@chph.ras.ru (A.A.)

**Keywords:** plasmon photocatalysis, electron injection, gold nanoparticles, antibacterial effects, biofilms, DNA repair, genes expression, quorum sensing, porins

## Abstract

In the present work, we provide evidence for visible light irradiation of the Au/TiO_2_ nanoparticles’ surface plasmon resonance band (SPR) leading to electron injection from the Au nanoparticles to the conduction band of TiO_2_. The Au/TiO_2_ SPR band is shown to greatly enhance the light absorption of TiO_2_ in the visible region. Evidence is presented for the light absorption by the Au/TiO_2_ plasmon bands leading to the dissolution of Au nanoparticles. This dissolution occurs concomitantly with the injection of the hot electrons generated by the Au plasmon into the conduction band of TiO_2_. The electron injection from the Au nanoparticles into TiO_2_ was followed by femtosecond spectroscopy. The formation of Au ions was further confirmed by the spectral shift of the transient absorption spectra of Au/TiO_2_. The spectral changes of the SPR band of Au/TiO_2_ nanoparticles induced by visible light were detected by spectrophotometer, and the morphological transformation of Au/TiO_2_ was revealed by electron microscopy techniques as well. Subsequently, the fate of the Au ions was sorted out during the growth and biofilm formation for some selected Gram-negative bacteria. This study compares the bactericidal mechanism of Au ions and Ag ions, which were found to be substantially different depending on the selected cell used as a probe.

## 1. Introduction

TiO_2_ photocatalysis induced by UV light possess powerful antimicrobial properties against bacteria, including multiresistant bacterial strains, viruses, and fungi. TiO_2_ photocatalysts need UV-light photons with energies ≥3.2 eV. The photogenerated electron–hole pairs lead to the generation of reactive oxygen species (ROS) in aqueous solution, leading to the degradation of contaminants and microorganisms [1,2]. Photocatalytic systems with biocidal effects under visible light are have not been widely reported and their mechanism of bacterial inactivation is only partially understood [3]. The photocatalytic/biocidal activity of Au/TiO_2_ nanocomposites is higher compared to bare TiO_2_ when excited by UV light. Electrons and holes are generated in TiO_2_ due to the interband transition induced by the UV quanta absorption. Au promotes the electron/hole separation and leads to ROS formation [4,5].

Controversial results about the cytotoxicity of gold nanoparticles (Au-NPs) have been reported [6,7,8]. Antibacterial activity of Au nanoparticles against several bacteria has been suggested for *E. coli* and the *Bacillus* Calmette–Guérin antituberculosis strain of attenuated live bovine tuberculosis *Bacillus* (*Mycobacterium bovis* BCG) [8,9], and *Staphylococcus aureus* [10,11]. It has also been reported that spherical Au nanoparticles with a variety of surface modifiers were not inherently toxic to cells under dark conditions [11,12]. Some chemicals used as precursors of the Au nanoparticles have been reported to induce toxicity. In this case, the Au-NPs themselves did not lead to bacterial inactivation [11].

Uncoated Au-NPs present a low antibacterial activity under visible light due to surface plasmon resonance (SPR). Under low light intensity, the Au-NPs’ heating was negligible [13]. Activity of Au-NPs becomes higher under light-emitting diode (LED) light providing a higher intensity radiation [14]. In parallel to the ROS production, Au nanoparticles activated by light may induce a photothermal bacterial inactivation as an additional effect [15,16,17,18].

The excitation of localized surface plasmons in Au-NPs generates hot electrons that transfer to the TiO_2_ conduction band and significantly enhance the light-harvesting capabilities of the photocatalytic system [19,20]. The Au ions, as well as ions of other heavy metals, have been reported to lead to bacterial inactivation [21,22]. The mechanism of antibacterial action of Au ions on bacterial cells have scarcely been studied. The antibacterial activity of Au ions began to be used in medicine after the discovery of Robert Koch showing that gold cyanide presented bacteriostatic effects on tuberculosis pathogens. Au complexes have been investigated for the treatment of rheumatoid arthritis and cancer (more specifically, cytotoxic effect against melanoma and leukemia cells) [22,23,24,25]. The goal of the present work is to study the photocatalytic bactericidal effect under visible light due to the Au/TiO_2_ nanoparticles’ surface plasmon resonance band (SPR). This effect leads to electron injection from the Au nanoparticles to the conduction band of TiO_2_, leading to the generation of Au ions. The antimicrobial effect of Au/TiO_2_ nanoparticles induced by visible light was observed to be significant. This work investigates the effect of Au ions on the growth and biofilm formation of some Gram-negative bacteria. The data obtained in the present work indicate significant differences in the mode of interaction of Au ions compared to Ag ions on bacterial cells [26].

## 2. Materials and Methods

### 2.1. Preparation of Au/TiO_2_ and Au Nanoparticles

The deposition of Au nanoparticles on TiO_2_ (10 g/L) was carried out by photocatalytic reduction of Au^3+^ ions in aqueous solution of HAuCl_4_ (1 mM, Sigma-Aldrich Chemie GmbH, Steinheim, Germany). Methanol was added to the solution (8% volume) as a hole scavenger [19]. The suspension was irradiated for 20 min by an ultraviolet fluorescent lamp (wavelength 365 nm, intensity 50 mW/cm^2^, Philips, Amsterdam, Dutch) under stirring. The Au/TiO_2_ nanoparticles were washed away from the synthesis products for further experiments. The suspension was transferred to an Eppendorf tube and centrifuged for 15 min at 7000 rpm until complete precipitation of the particles. The solution above the precipitate was separated and the precipitate of Au/TiO_2_ nanoparticles washed 15 times.

Au nanorods were synthesized according to the protocol reported by [27]. The details of the synthesis are presented in the Supplementary Information (Section SI1). The suspension of Au-NPs was centrifuged for 15 min at a speed of 7000 rpm until the precipitation of the particles was completed. The supernatant solution above the precipitate was separated and the precipitate washed for 15 times. The Au nanoparticles were suspended in a solution of PEG4000 (0.1 g/mL Merck KGaA, Darmstadt, Germany). A scanning electron microscope (SEM) image of the Au nanoparticles is presented in Figure SI1.

The standard method of spherical Au-NP synthesis was used with some modifications [28]. The reduction of a HAuCl_4_ solution was initiated by sodium tris-citrate (Sigma-Aldrich Chemie GmbH, Steinheim, Germany) and the solution was heated to the boiling temperature under stirring (SI1). When the solution (147 μL of 0.2 M HAuCl_4_ in 100 mL water) reached 95 °C, the citrate solution (3 mL, 0.034 M) was added. After 20 min, the liquid was extracted and cooled to room temperature. A SEM image of the Au nanoparticles is present in Figure SI2.

### 2.2. Irradiation Procedures on the Au/TiO_2_, Electron Microscopy, and Laser Femtosecond Experimental Setup

The photocatalytic effect of the Au/TiO_2_ nanoparticles due to surface plasmon resonance (SPR) excitation was studied under the light of a light-emitting diode (LED, wavelength 530 nm, intensity 30 mW/cm^2^, Nichia, Tokushima, Japan). The bacterial inactivation runs of the bacterial cells were run in an Au/TiO_2_ suspension under a tungsten–halogen lamp: J118 500W R7s Phoenix (Linyi Yuhang Lighting Electric Appliance Co, Ltd, Shandong, China). The tungsten–halogen lamp spectrum is shown in the Supplementary Information Section SI2, Figure SI3). A cutoff infrared water filter (20 cm length, homemade) was used. The UV component of the light was blocked by a colored glass filter with a cutoff at 440 nm. The intensity of light reaching the sample was 60 mW/cm^2^.

#### 2.2.1. Electron Microscopy

The SEM images of Au-NPs and Au/TiO_2_ nanoparticles were obtained with: (1) a Zeiss G25-SUPRA 25en01 (Carl Zeiss AG, Jena, Germany); (2) Carl Zeiss NVision 40, equipped with an Oxford X-Max X-ray detector. A voltage of 1 kV was used. The magnification of the micrographs ranged from 3000× to 300,000×. X-ray microanalysis was carried out at an accelerating voltage of 20 kV. Transmission electron microscope was a LEO 912 АВ Omega (Carl Zeiss AG, Jena, Germany) with LaB6 cathode at a voltage of 100 kV.

#### 2.2.2. Femtosecond Laser Photolysis Setup

A femtosecond pump (Spectra-Physics, Santa Clara, CA, USA)—supercontinuum probe setup was used to register the transient absorption (TA) spectra of the Au/TiO_2_ colloids as reported recently [29]. The femtosecond pump pulse of 25 fs centered at 740 nm (1.68 eV) and a low fluency of 24 μJ/cm^2^ was used to excite the Au/TiO_2_ nanoparticles in solution. The pump and probe light spots had the diameters of 300 and 120 µm, respectively. The measured TA spectra were corrected for group delay dispersion of the supercontinuum using a protocol described previously [29]. Details of the femtosecond setup are shown in the Supplementary Information, Section SI3.

### 2.3. Preparation of Bacterial Strains, Evaluation of the Bacterial Inactivation Kinetics, and Bacterial Growth Conditions

Minimum inhibitory concentration (MIC) values for different strains of Gram-negative bacteria are shown in Table 1. Strains were maintained on Luria–Bertani broth solid agar (1.5% agar, LA, Sigma-Aldrich Chemie GmbH, Steinheim, Germany) at 30 °C. Difco Nutrient Broth (NB) and Difco Nutrient Agar (NA) were used in experiments with AuHCl_4_. Antibiotics were added to the medium in the following concentrations (µg/mL): ampicillin (Ap, OAO “Biochemist”, Moscow, Russia), 100; kanamycin (Km, OAO “Synthesis”, Moscow, Russia), 100; tetracycline (Tc, Sigma-Aldrich Chemie GmbH, Steinheim, Germany), 20–40, gentamicin (Gm, KRKA, Novo mesto, Slovenia), 40; and rifampicin (Rif, Sigma), 50. The chemicals used during this study: HAuCl_4_·3H_2_O (99.9%), NaBH_4_ (99%), ascorbic acid (99%), cetyl-trimethyl-ammonium bromide (CTAB, 99%), trisodium citrate dihydrate, AgNO_3_ (99%), and poly(ethylene glycol) PEG 40,000, were used as purchased (Sigma-Aldrich). TiO_2_ Aeroxide^®^ P25 (Degussa P25, Frankfurt, Germany) was used as received.

#### 2.3.1. Minimum Inhibitory Concentrations (MICs) and Biofilm Formation

The MICs of AuHCl_4_ were measured by serial twofold dilution [26]. The initial bacterial solution colony-forming units CFU/ml was ~1 × 10^5^ in nutrient broth (NB). The concentration of planktonic cells was evaluated by optical density at 600 nm (OD_600_). The measurements were made after the incubation of bacteria for 24 h at 30 °C.

#### 2.3.2. Biofilm Formation

The monitoring of the film formation was carried out following the protocol described by Zaitseva et al. [30]. Cells from fresh LA medium were inoculated into NB and incubated under shaking for 24 h at 30 °C. The culture was then diluted 300-fold in fresh NB, adding selected AuHCl_4_ concentrations. Inoculated cultures were grown in 96-well polystyrene microtiter plates (150 µL per well) for 24 h at 30 °C under gentle shaking (105 rpm) to prevent the formation of sediment at the bottom of the wells. The growth of planktonic (unattached) cells was evaluated by measuring the optical density at 600 nm (OD_600_) of the cell suspension. Biofilm formation was measured by discarding the medium, rinsing the wells with distilled water, and staining the attached cells with 1% w/v crystal violet (Reachim, Moscow, Russia). After staining for 45 min at 24 °C, the supernatant was then removed from the flask and the wells were rinsed three times with distilled water. Then, the biofilm-associated crystal violet was solubilized with ethanol and the absorbance measured at 600 nm. A microplate reader (Model 2550 Microplate Reader, Bio-Rad, Hercules, CA, USA) was used for measuring planktonic growth and biofilms. The mean CFU/ml for negative untreated samples were: ~1.8 × 10^8^; 2.2 × 10^8^; 3.0 × 10^8^ for *E. coli* AB1157, *P. aeruginosa* PAO1, and *S. proteamaculans* 94, respectively.

A bacterial growth time of 24 h was found to be optimal for biofilm formation. Experiments were repeated four times and bacteria were grown in eight replicate wells during each experiment. A study of cultures from wells under a microscope revealed no cell aggregates. The cell suspensions were turbid, which precludes the formation of aggregates. No biofilms were observed on the surface of the wells. Biofilms were formed on the well walls at the border of the medium surface and the air and not at the bottom of the wells.

#### 2.3.3. Effect of AuHCl_4_ on the *E. coli* AB1157 Biofilm and Effect of Au Ions on Bacterial Survival

An overnight culture of *E. coli* AB1157 was diluted 300-fold in NB, and thereafter the cells were grown on glass slides for 24 h at 30 °C, leading to the formation of biofilms. Then, the supernatant excess solution was removed from the slides and a fresh NB solution was added and supplemented with AuHCl_4_. The bacterial cells were further grown for 24 h. Biofilms were analyzed by using crystal violet as described above. Bacterial viability in biofilms was assessed with Live/Dead BacLight Bacterial Viability and Counting Kit (Invitrogen, Molecular Probes) via scanning confocal laser microscopy (Zeiss LSM 510 laser module, Carl Zeiss GmbH, Jena, Germany) with an excitation wavelength at 488 nm.

#### 2.3.4. Effect of Au Ions on *E. coli* AB1157 Cell Survival in Stationary and Exponential Growth Phases

An overnight culture of *E. coli* AB1157 was diluted 100-fold in NB, and then the cells were grown for 4 h at 30 °C under shaking. The number of cells in different variants of the experiment were equalized: the effects of AuHCl_4_ on bacterial cells were compared by way of overnight culture diluted 10 times in NB (stationary phase) and a culture was grown in NB for 4 h (exponential phase). AuHCl_4_ was added to both cultures and the solutions were shaken for 2 h. Then, the cells were plated on NA medium with 1.5% agar.

#### 2.3.5. Statistical Analysis

The results related to MIC and biofilm characterizations were expressed in terms of the mean of *n* numbers of independent experiments standard deviation (SD). The data were analyzed using Microsoft Excel software.

## 3. Results and Discussions

### 3.1. Photocatalytic Bactericidal Effect and Femtosecond Laser Spectroscopy of Au/TiO_2_ Suspensions

**Figure 1:**
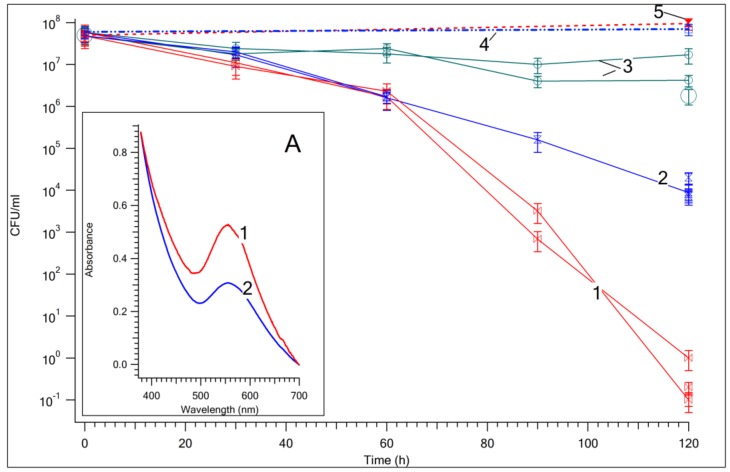
CFU/ml plotted as a function of *E. coli* AB 1157 inactivation time for solutions: 1, Au/TiO_2_ suspension irradiated under visible light; 2, TiO_2_ suspension irradiated under visible light; 3, water irradiated under visible light; 4, Au/TiO_2_ suspension in dark; 5, TiO_2_ suspension in dark. Insert A: spectrum 1, absorption spectrum of Au/TiO_2_ before visible light irradiation; spectrum 2, absorption spectrum of Au/TiO_2_ after 2 h under visible light in nutrient broth (NB).

Figure 1 shows the CFU decay of *E. coli* AB1157 in aqueous solution photocatalyzed by suspensions of TiO_2_ and Au/TiO_2_ in the dark and also under visible light irradiation. Bacterial inactivation in the dark was not observed in Au/TiO_2_ and TiO_2_ suspensions. This indicates that Au/TiO_2_ and TiO_2_ are not intrinsically toxic towards *E. coli* AB1157. Moderate bacterial inactivation kinetics were induced by TiO_2_, as shown in Figure 1 (trace 2), due to the TiO_2_ surface states’ excitation under visible light irradiation [31]. A higher acceleration in the *E. coli* AB1157 CFU bacterial inactivation kinetics is seen in Figure 1 (trace 1) for suspensions of Au/TiO_2_ under visible light, as compared to TiO_2_. Heating of Au-NPs was estimated to not exceed 1 °C (see Supplementary Information, Section SI4). Measurement of the temperature of the Au/TiO_2_ suspension under visible light showed a heating of no more than 2 °C. Acceleration of the death of bacteria cannot be associated with the thermal effect. The insert in Figure 1 shows the absorption spectra of the Au/TiO_2_ suspension. The absorption bands with maximum at ~550 nm of the Au/TiO_2_ spectra correspond to the plasmon band of the Au-NPs in the nanocomposite (see insert A of Figure 1).

**Figure 2:**
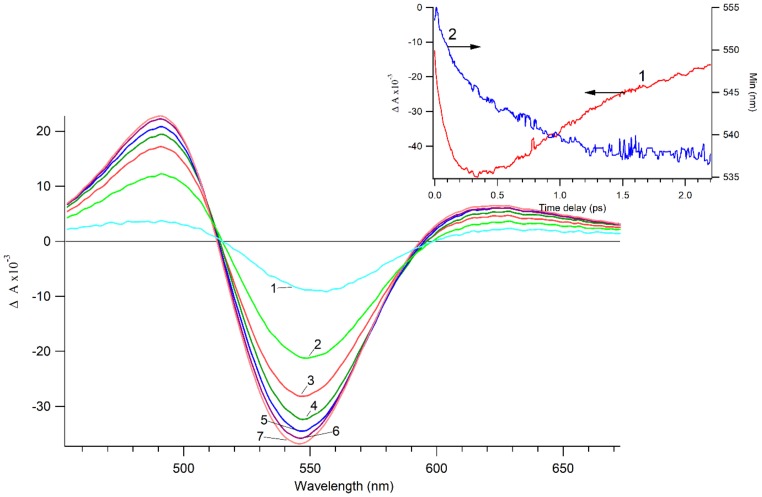
Transient absorption spectra Δ*A* (λ) of Au/TiO_2_ suspension after excitation by a 25-fs laser pulse at 740 nm. Time delay: 1, 0 fs; 2, 50 fs; 3, 100 fs; 4, 150 fs; 5, 200 fs; 6, 250 fs; 7, 350 fs. Insert: Trace 1: the left y-axis shows the amplitude of the plasmon bleaching band; Trace 2: the right y-axis shows the wavelength of the minimum of the plasmon bleaching band.

Figure 2 demonstrates the transient absorption spectra of Au/TiO_2_ suspension at a time delay shorter than 400 fs. The transient spectra consist of a bleaching band where a differential absorbance is negative Δ*A* < 0 and an absorption band where a differential absorbance is positive Δ*A* > 0. The bleach band position is close to maximum of the plasmon band. The appearance of the bleach band and the long wavelength absorption band is due to the shift and broadening of the plasmon resonance band in the photoexcited Au-NPs, due to the heating of the electron gas in the Au-NPs during the plasmon decay. This involves formation of a nonequilibrium electron and a subsequent thermalization process. The transient curve of the bleaching band amplitude shown in the insert of Figure 2 reveals the dynamics of the electron thermalization dynamics. Insert of Figure 2 shows the significant shift of the bleach band position that cannot be explained by the electron heating [19]. If the permittivity of the TiO_2_ in the Au/TiO_2_ NPs is changed, the position of the plasmon band is also shifted. Electrons injected from Au-NPs into TiO_2_ lead to an increase in the TiO_2_ refractive index (*n*_TiO2_) and permittivity ε_TiO2_ = (*n*_TiO2_)^2^ [19]. The change of permittivity can be a reason for the strong spectral shift of the plasmon band. Since the maximum position of the plasmon band is determined by the Fröhlich condition: Re(ε_Au_(ω)) = −2ε_m_, the increase in the medium permittivity is due to the increase of ε_TiO2_. This is associated with: (a) the long-wavelength shift of the plasmon band after Au-NP excitation and (b) the corresponding short-wavelength shift of the bleaching band seen in Figure 2. The dissolution of Au-NPs from the Au/TiO_2_ surface occurs concomitantly with the transfer of electrons generated by Au plasmon into the conduction band of TiO_2_. Under visible light, the Au-NP valence band holes (v_b_h^+^) give rise to Au ions that are released to the surrounding media. Electron injection from Au-NPs into TiO_2_ agrees with a recent study by Tian and Tatsuma [32]. The antibacterial effects of these Au ions in solution will be addressed in detail in the sections below.

**Figure 3:**
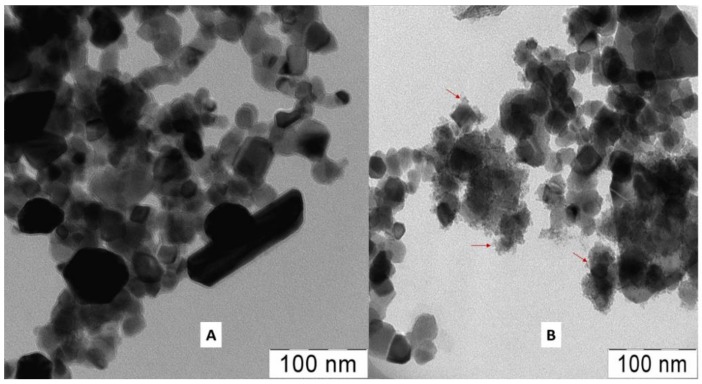
Images of Au/TiO_2_ nanoparticles before and after 24 h irradiation with LED green light (300 μW/cm^2^). (**A**) Au/TiO_2_ nanoparticles before irradiation; (**B**) Au/TiO_2_ nanoparticles after irradiation. Arrows indicate fine formations. LED: light-emitting diode.

Transmission electron microscopy images of Au/TiO_2_ NPs before and after 24 h irradiation (see Figure 3) with LED green light (300 μW/cm^2^) show that irradiation leads to the creation of chemical species associated with the fragmented Au-NPs. Bright- and dark-field transmission electron microscopy (TEM) images are shown in the Supplementary Information, Section SI5 (Figure SI4). This NPs fragmentation was not detected under similar visible light irradiation of pure TiO_2_. This observation agrees with the decrease of the plasmon band amplitude shown in the insert of Figure 1. Elemental analysis of Au/TiO_2_ nanoparticles before and after 24 h irradiation shown the decrease of the Au in the Au/TiO_2_ powder. This confirms the Au dissolution due to the surface plasmon resonance in the Au/TiO_2_ bands.

### 3.2. MICs of AuHCl_4_ for Different Gram-Type Bacteria and Effect of AuHCl_4_ on the Biofilm Formation

#### 3.2.1. MICs of AuHCl_4_ of Different Gram-Type Bacteria

The bacteria cells for the determination of MICs were cultured in NB medium. The MIC values of AuHCl_4_ measured for Gram-negative bacteria (*E. coli* AB1157, *P. aeruginosa* PAO1, and *S. proteamaculans* 94) are summarized in Table 1. The MIC values for *E. coli* AB1157 and *S. proteamaculans* 94 were close, and the resistance of *P. aeruginosa* PAO1 to AuHCl_4_ was much higher. We examined effect of Au nanoparticles on *E. coli* AB1157 cells. Spherical Au nanoparticles and Au nanorods did not inactivate *E. coli* AB1157 cells. As some precursors of nanoparticles, such as the detergent CTAB, might induce toxicity, the nanoparticles themselves are not necessarily detrimental to cellular function (see Section SI6). This result agrees with conclusions made by Connor et al. [11].

#### 3.2.2. Effect of AuHCl_4_ on the Formation of Biofilms

**Figure 4:**
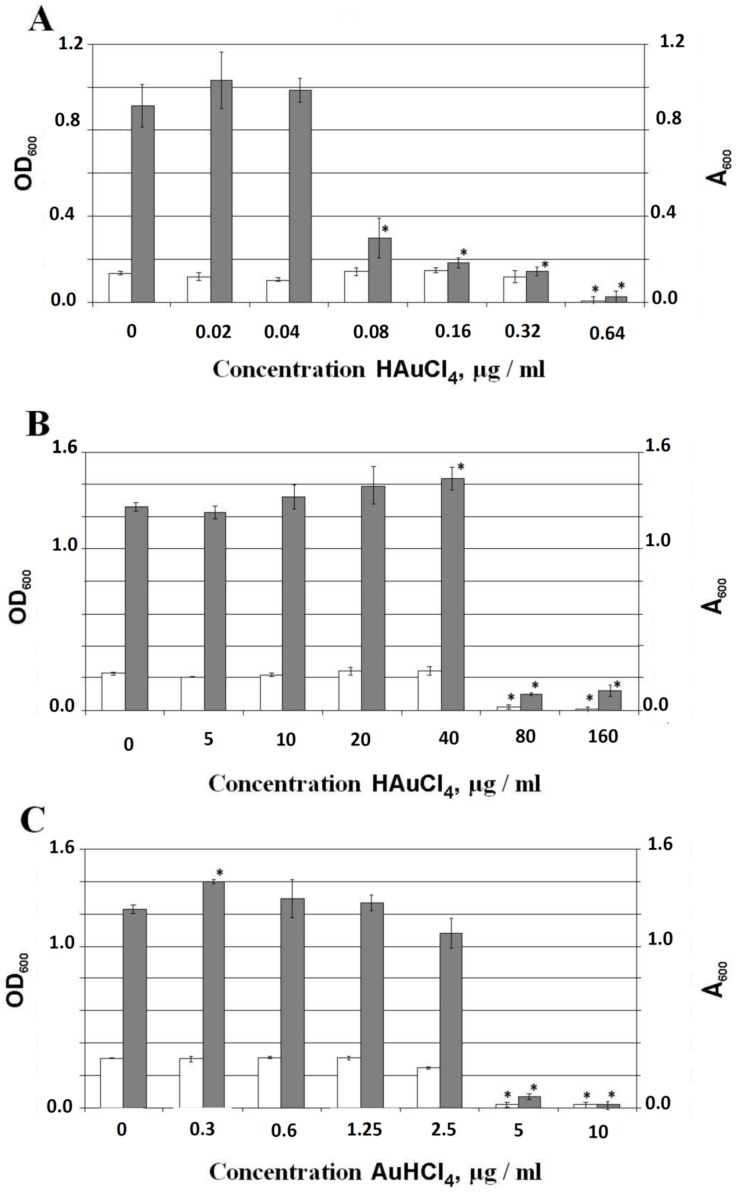
Effect of AuHCl_4_ on biofilm formation and planktonic cells. (**A**) *E. coli* AB1157. (**B**) *P. aeruginosa* PAO1. (**C**) *S. proteamaculans* 94. OD_600_, optical density of planktonic cells; A_600_, absorption of crystal violet staining of biofilms. White bars refer to planktonic growth; dark bars refer to biofilms. Errors were determined from a set of 4 measurements with 8 replicate samples. The standard deviation is shown in the figures. According to the Student’s *t*-test (*p* < 0.05), the bars marked with an asterisk (*) are significantly different from the negative (untreated) control (0 μg/mL of AuHCl_4_). The mean CFU/ml for negative (untreated) controls were: ~1.8 × 10^8^, 2.2·× 10^8^, and 3.0·× 10^8^ for *E. coli* AB1157, *P. aeruginosa* PAO1, and *S. proteamaculans* 94, respectively.

Figure 4 shows the effect of AuHCl_4_ on the planktonic growth (OD_600_) and biofilm formation evaluated by the absorbance of crystal violet staining of biofilms of *E. coli* AB1157, *P. aeruginosa* PAO1, and *S. proteamaculans* 94. Planktonic growth was practically absent at a concentration of AuHCl_4_ of 0.64 µg/mL for *E. coli* AB1157, 80 μg/mL for *P. aeruginosa* PAO1, and 5 μg/mL for *S. proteamaculans* 94. The decrease of the bacterial mass in the biofilm was detected by the A_600_ absorbance. This was observed when the AuHCl_4_ concentration was > 0.04 µg/mL for *E. coli* AB1157, 40 μg/mL for *P. aeruginosa* PAO1, and 2.5 μg/mL for *S. proteamaculans* 94. OD_600_ of the biofilm biomass decreased almost to zero at AuHCl_4_ concentrations of 0.64 μg/mL for *E. coli* AB1157, 10 μg/mL for *S. proteamaculans* 94, and >160 μg/mL for *P. aeruginosa* PAO1.

#### 3.2.3. Effect of AuHCl_4_ on Mature Biofilms of *E. coli* AB1157

**Figure 5:**
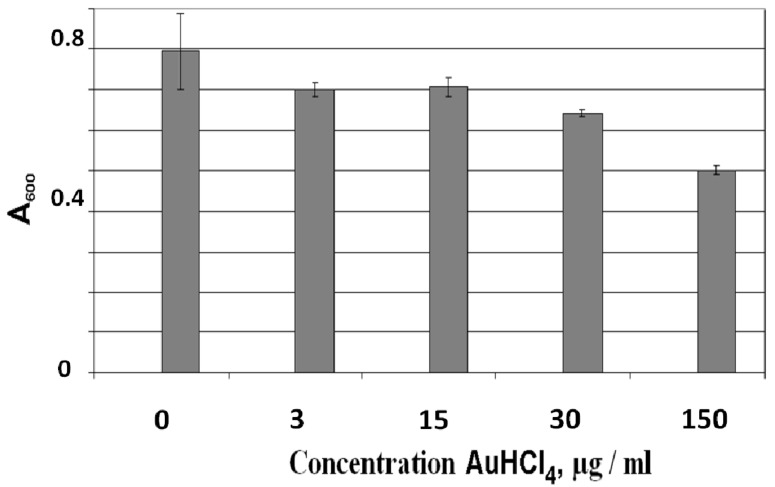
Effect of AuHCl_4_ concentration on the mature biofilms. A_600_ is the absorption of crystal violet-stained biofilms, which is a measure of the biofilm biomass. Errors were determined from a set of 4 measurements with 8 replicate samples.

**Figure 6:**
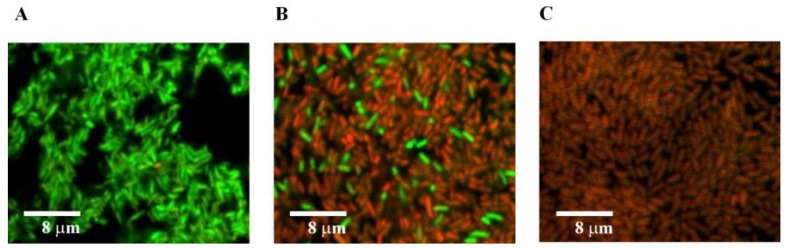
Confocal microscopy of *E. coli* AB1157 biofilms. (**A**) Confocal microscopy of *E. coli* AB1157 cells at 0 μg/mL AuHCl_4_; (**B**) at 15 μg/mL; (**C**) at 150 μg/mL AuHCl_4_. Viable cells are shown by green color and dead cells by red.

The biofilms were grown in NB on glass slides under static conditions (without shaking). After biofilm growth for 24 h, the culture of planktonic cells was removed and a fresh NB solution containing AuHCl_4_ up to 150 µg/mL was added. Analysis of the biofilms with crystal violet staining showed that the decrease in the biofilms began at an AuHCl_4_ concentration of 3–15 μg/mL. An increase in the AuHCl_4_ concentration led to a further decrease of the biofilm biomass (Figure 5). Figure 6 presents the confocal microscopy images of *E. coli* AB1157 biofilms without AuHCl_4_ treatment (a, control, green cells) and after the treatment by 15 µg/mL (b) and 150 µg/mL (c) AuHCl_4_. The treatment of biofilms with 150 µg/mL of AuHCl_4_ led to cell death in the biofilms (red cells). This shows that the AuHCl_4_ concentrations completely inhibiting biofilm formation of *E. coli* were insufficient to kill bacterial cells in mature biofilms. The existence of bacteria in biofilms protected them from the action of AuHCl_4_. Figure 6 shows that *E. coli* cells died at an AuHCl_4_ concentration of 150 µg/mL. However, at a similar AuHCl_4_ concentration, the biofilm biomass decreased by only ~1.6 times from A_600_ 0.8 to 0.5 (see Figure 5). This shows that the matrix biofilm mass was less reduced compared to the living cells in the biofilm.

### 3.3. The Effect of Phase of Growth on the Sensitivity of E. coli AB1157 Cells to AuHCl_4_

To clarify the increased resistance of bacterial biofilms to the action of Au ions, the survival of *E. coli* cells in the presence of AuHCl_4_ was investigated at different growth stages. The high density of bacteria in biofilms, the lack of access to nutrients and oxygen, and metabolite accumulation of the bacteria inside the biofilms are similar to those in the physiological state with the cells in a stationary phase or in a phase of growth retardation. Since the bacterial cells in a stationary growth phase are usually more resistant to unfavorable external effects, it is assumed that the physiological state of cells living in biofilms led to the cells’ high resistance. The influence of the growth phase on the sensitivity of bacteria to Au compounds has not been reported. We showed that resistance to the action of AuHCl_4_ of *E. coli* AB1157 cells in the early stationary phase of growth was higher than that in the exponential-phase cells (see Table 2).

### 3.4. About the Effect of the Au Ions’ Interaction with Bacteria

Earlier work on the mechanisms of silver ions’ and silver nanoparticles’ interaction with bacterial cells [26] demonstrated that the increased cell sensitivity to the silver ions/NPs was due to mutations in the genes responsible for DNA repair of the oxidative damage (*mut*Y, *mut*S, *mut*M, *mut*T, *nth*). We have assumed on the basis of this data that these genes are involved in the restoration of DNA oxidatively damaged by silver compounds. Because cellular DNA could be damaged by AuHCl_4_, it was interesting to determine if the DNA repair is involved in the protection of cells from Au ions. For this purpose, we compared the AuHCl_4_ MICs for isogenic *E. coli* wild-type strains and mutant strains deficient in different repair systems. The data is presented in Table 3. There is no information at the present time in the available literature regarding the participation of DNA repair systems in the protection of bacterial cells toward Au ions. Upon comparison of the sensitivity to Au ions of cells of *E. coli* BW25113 (wild-type strain) and a number of mutants deficient in proteins involved in oxidative DNA repair (*mut*Y, *mut*S, *mut*M, *mut*T, *nth*), no significant differences were found between these strains (see Table 3). The slight increase in sensitivity to the Au ions was observed in the bacterial cells with the mutations in the *mut*S and *mut*Y genes. Enzymes encoded by these genes remove adenine bases that are misincorporated opposite to 8-oxoG (8-oxoguanine, the most common oxidative lesion) [33,34,35]. The data shows that the sensitivity of cells to Au ions is not significantly related to oxidative DNA damage by Au ions. Mutant strains deficient in SOS repair genes (*rec*A, *lex*A, *umu*C, *umu*D) showed practically the same sensitivity to AuHCl_4_ as the wild-type strains (see Table 3). This means that the genes involved in DNA repair are not important for the bacteria cell resistance to AuHCl_4_. Mutation in the *uvr*A6 gene did not increase significantly the sensitivity of *E. coli* to Au ions. It is known that the global regulator sigma S subunit of RNA polymerase encoded by *rpoS* controls the transcription of a large number of genes under various types of stress. Genes activated by sigma S encode proteins that protect cells from stress [36]. It is expected that cells harboring the *rpoS* mutation would be more sensitive to the action of AuHCl_4_. However, the sensitivity to Au ions of cells presenting this mutation was almost unchanged. Porins are the proteins of the outer membrane in Gram-negative bacteria present in the water-filled channels enabling the influx and efflux of low-molecular-weight compounds through the outer membrane of the bacterial cell [37]. *E. coli* mutants deficient in OmpF and/or OmpC porins were 4–8 times more resistant to Ag ions. This validates their important role in the transport of silver ions to the bacterial cell [26,38,39]. Mutant strains lacking porins OmpF or OmpC did not differ significantly in their sensitivity to Au ions as compared to the wild-type strain. The mechanism of bactericidal action of Au ions and Ag ions on bacterial cells are substantially different.

### 3.5. The Effect of the Au Ions’ Interaction with Bacteria

Only a few reports have addressed the effect of Au ions on bacterial cells [22]. As noted in the preceding sections in this study, cells in mature biofilms are more resistant to drugs/antibiotics and unfavorable environmental factors when exposed to their effects than planktonic cells. Only a few reports are available reporting data on the resistance of biofilms to silver ions and silver NPs, or on the effect of the later compounds on the growth/biofilm formation by planktonic cells. During the course of this work, we did not find data about the effect of Au ions on the formation of biofilms and mature biofilms in the literature. This motivated us to investigate this problem. The mechanism of the high resistance of bacteria observed in biofilms is extremely important in the field of antibiotic therapy.

During this work, we found that Au ions inhibit biofilm formation and cause cell death on preformed biofilms. The cell death in mature biofilms was observed at higher concentrations of Au ions compared to the Au concentration required during the film formation stage. Bacteria in biofilms were up to 150 times more resistant to the action of AuHCl_4_ than planktonic cells. The increased resistance of bacterial cells in biofilms has been reported for the salts of copper, lead, zinc [40], silver ions, and silver nanoparticles [26,41]. The biofilm resistance has been reported to depend on the penetration of antimicrobial agents in the biofilm matrix, the metabolic activity and growth rate of biofilm bacteria and planktonic growing cells, and on the population of persistent cells able to survive stress conditions [41,42]. The physiological condition and the metabolic activity of cells living in biofilms are closer to those in the stationary phase than the cells in the growth stage. This is one reason for the increased cell resistance to unfavorable factors. It is known that bacteria in the growth phase of growth retardation and in the stationary phase are more resistant to antibacterial agents. This is important in relation to antibiotics targeting cell division. One study reported that the increased resistance of bacteria in biofilms involves a multifactorial process [43].

The resistance of bacteria cells to Au ions within the growth phase has not been reported. The present study shows that that the *E. coli* cells in the growth phase were significantly more resistant to Au ions than cells in the logarithmic phase. Thus, it can be assumed that the increased cell resistance in biofilms to AuHCl_4_ is due to the physiological state of the bacteria inside the biofilm being similar to the state of the cells in the stationary growth phase. We did not obtain data about participation of DNA repair systems in the protection of the bacterial cells against Au ions. There were no significant differences in the sensitivity of bacteria to Au ions in comparison with wild-type strains and mutant strains deficient in SOS repair (*rec*A, *lex*A, *umu*C, *umu*D), excision repair (*uvr*6) or DNA repair of oxidative damage caused by ROS (reactive oxygen species) (*mut* S, *mut*Y, *mut*M, *mut*T). These data suggest that the sensitivity of bacteria to Au ions is not related to DNA damage that can be recovered by way of DNA repair systems. It can be assumed that the effect of Au ions on the bacterial cells is mainly due to the binding of Au ions to the DNA bases. This interaction has been reported to be important in the case of silver ions and preferably occurs with GC pairs of the DNA, leading to serious damage [44,45]. However, detailed study of DNA damage due to Au ions was not carried out during the course of this study. Cell mechanisms that may be involved in the repair of cell damage is also lacking at the present time.

It has been shown that mutant strains with inactivated genes encoding the transport proteins porins did not differ significantly in their sensitivity to Au ions compared to the wild-type strain. Thus, it can be concluded that porins are not involved or only partially involved in the transport of Au ions in the cells. This is not the case for the transport of silver ions [26]. This observation suggests the possibility of other mechanisms taking place during the Au ion penetration into the cells. The removal of metal by means of active transport through transporter proteins from the cytoplasm to the periplasmic space is well-known to be involved with bacterial resistance to metals [43]. Binding of Au ions on the cell membrane is also possible, similar to the adsorption of other heavy metal ions [46].

### 3.6. Benefits of the Information Reported in This Study: Outlook and Future Work

Healthcare-associated infections (HCAIs) present a major public health problem worldwide. Hospitals are a reservoir of pathogenic microorganisms, leading to a higher incidence of HCAIs. During the last few years, an increase in HCAIs caused by mutant pathogen strains, especially drug-resistant strains, has been noticed in hospitals. Occurrence of infections resistant to both the immune system and antibiotics has increased during the last decade. Vancomycin and gentamicin are used in combating infections caused by biofilms on tissues and implants, but their action does not exceed a few days and is irregular.

Biofilms are the most dangerous type of bacterial presence, spreading infections over long time periods, and this is the reason why some aspects of the Au/TiO_2_ interaction with biofilms have been documented in a quantitative way and presented in detail in this study. More advanced antimicrobial materials at this stage are necessary, and Au/TiO_2_ releasing low-cytotoxicity Au ions is shown to present antimicrobial activity with advantages compared to antibiotics. The later compounds promote the development of bacterial resistance when administered over longer treatment periods. This study has shown that Au/TiO_2_ NPs disinfect bacteria by releasing adequate amounts of Au ions while keeping sufficient reserves to ensure long-term operational disinfection. Metals can eliminate/destroy more than 99% of the microbial strains, and antibiotics coupled with Au ions are not available at the present time. They could improve the present situation due to the small size of the Au ions enabling them to penetrate tissues not reached adequately by antibiotics. This study shows that Au ions in ppb concentrations lead to effective disinfection. This information may be used to assess if the concentrations of Au- ions leading to bacterial disinfection are below the guidelines imposed by sanitary regulations. This study presents basic information that can be useful for the preparation of Au-NPs on other substrates than TiO_2_ to increase selectivity, giving high-quality functionalized Au coatings that reduce the toxicity to mammalian cells, while maintaining antimicrobial activity and a high biocompatibility. The development of Au/TiO_2_ NPs activated by solar/visible/actinic light is in its infancy.

The bacterial cell membrane is both a barrier and a channel for the inward and outward movement of chemical species with sizes below the porin diameters. In the Gram-negative bacterial membrane structure, porins allow the passage of molecules <600 Da in and out of the bacteria. The small size of the metal ions allows the Au ions to enter the pathogen and translocate through the bacterial cell wall. However, we do not know the fate of the intracellular Au/TiO_2_ NPs or Au ions. This is still a controversial issue and cannot be yet answered. The problem is that the majority of cell culture studies are done under suspension, and thus then difficult to differentiate between the particle and ionic effects.

This study presents the interfacial interaction between Au/TiO_2_ and bacteria in detail and suggests the mechanism leading to bacterial inactivation. However, the full bactericidal inactivation mechanism is still not understood. The Au/TiO_2_ NPs exhibit promising antibacterial kinetics and offer a potential new approach for the effective prevention of bacterial infections by pathogenic biofilms, as investigated during the course of this study. The benefit of establishing the Au disinfection mechanism is that this is a necessary step for any commercial application of a light-induced antibacterial process.

## 4. Conclusions

This work presents femtosecond dynamics of Au/TiO_2_ SPR plasmon-band decay leading concomitantly to the generation of toxic Au ions that subsequently induce bacterial inactivation in different bacterial strains. The effects AuHCl_4_ on the cell biofilm formation and planktonic cells is reported in a quantitative way. This study reports some aspects related to the effect of mutations in the genes responsible for DNA repair and on the sensitivity of *E. coli* to the Au ions in a detailed way. Au ions were found to inhibit biofilm formation and led to cell death in preformed biofilms. To achieve cell death in mature biofilms required higher Au ion concentrations compared to the Au concentrations needed for the biofilms investigated during the early formation stages.

This study also reports on the Au ions’ mechanism of interacting with the bacterial cells. This has not previously reported and was found to be different compared to the interaction with Ag ions. This study also reports on the effect of gene mutations responsible for DNA repair and on the sensitivity of *E. coli* to the Au ions.

## Figures and Tables

**Table 1:** Values for different strains of Gram-negative bacteria.

Strains	MIC of AuHCl_4_ (μg/mL)*
*Escherichia coli* AB1157	1.0 ± 0.3
*Pseudomonas aeruginosa* PAO1	80 ± 10
*Serratia proteamaculans* 94	1.4 ± 0.3

* The MIC values are a result of the data collected from 4 independent runs.

**Table 2:** The influence of phase of growth on survival of *E. coli* AB1157 in solutions with Au ions*.

AuHCl_4_ (μg/mL)	Exponential Phase of Growth (cells/mL)		Stationary Phase of Growth (cells/mL)	
0	16·× 10^7^	100% **	22·× 10^7^	100%
5	1.4·× 10^7^	8.8%	8.2·× 10^7^	38%
8.5	8·× 10^5^	0.5%	12.6·× 10^6^	6%
17	4.8·× 10^5^	0.3%	5·× 10^6^	2.3%

*Data from triplicate runs. ** Percentage of colonies in the control (without AuHCl_4_).

**Table 3:** AuHCl_4_ MICs of *E. coli* strains with deficiency in DNA repair, in global regulation of genes expression, and in porin synthesis.

Strains of *Escherichia coli* K12	Characteristics	MIC of AuHCl_4_, μg/mL*
SBS 1936SBS 2680	*rpo* S^+^ *rpo* S^−^	1.3 ± 0.21.0 ± 0.3
AB 1157AB 1886AB 2463AB 2494	*uvr*A^+^*uvr*B^+^*rec*A^+^*lex*A^+^*rec*BC^+^*rec*F^+^*lon*^+^ *uvr* A6 *rec* A13 *lex* A	1.0 ± 0.30.5 ± 0.20.7 ± 0.21.0 ± 0.3
MC 4100TK 821MH 1471MH 225	*omp*R^+^*omp*F^+^*omp*C^+^*omp*R^+^*omp*F^−^*omp*C^−^*omp*R^+^*omp*F^−^*omp*C^+^*omp*R^+^*omp*F^+^*omp*C^−^	1.0 ± 0.31.3 ± 0.31.0 ± 0.31.0 ± 0.3
BW 25113JW 2703JW 2928JW 3610JW 0097	*rrnB3* Δ*lacZ4787 hsdR514* Δ*(araBAD)567*Δ*(rhaBAD)568 rph-1mutS::kanmutY::kanmutM::kanmutT::kan*	1.0 ± 0.30.5 ± 0.20.5 ± 0.21.3 ± 0.31.0 ± 0.3
TK 603TK 610TK 612	*uvr* A6*umu* C36*umu* C44	1.0 ± 0.31.0 ± 0.30.8 ± 0.2

* Values of MICs are obtained from data from 5 independent runs.

## Supplementary Materials

The following are available online at http://www.mdpi.com/2079-4991/9/2/217/s1, S1: Synthesis of Au nanoparticles, S2: The spectrum of tungsten–halogen lamp, S3: Femtosecond laser photolysis setup, S4: The estimation of the local temperature at the surface of Au-NPs in an Au/TiO_2_ sample under steady-state illumination, S5: Bright- and dark-field TEM images of Au/TiO_2_ NPs, S6: The evaluation of bactericidal effect of residual solution after Au nanorod synthesis.
