# Supplementary material for: Femtosecond Spectroscopy of Au Hot-Electron Injection into TiO2: Evidence for Au/TiO2 Plasmon Photocatalysis by Bactericidal Au Ions and Related Phenomena
Source: Nanomaterials (Basel). 2019 Feb 6;9(2):217. doi: 10.3390/nano9020217 (PMC6410102; doi:10.3390/nano9020217)

**Au/$TiO_2$ plasmon photocatalysis: femtosecond spectroscopy of the hot electron injection into $TiO_2$, bacterial inactivation, bactericide Au-ions and related phenomena**

**M. A. Radzig [1], V. A. Plyuta[1], O. A. Koksharova [1,2], I. A. Khmel [1], V. K. Ivanov [3], K. E. Yorov [2,3], J. Kiwi [4], S. Rtimi [4], E. A. Tastekova [5], A. Aybush [5], V. A. Nadtochenko* [2,5].**

[1] Institute of Molecular Genetics, Russian Academy of Sciences, Kurchatov Sq. 2, 123182 Moscow, Russia
[2] A. N. Belozersky Institute of Physico-Chemical Biology and Chemical Department of M. V. Lomonosov Moscow State University, 119992 Moscow, Russia
[3] Kurnakov Institute of General and Inorganic Chemistry of the Russian Academy of Sciences, Leninskiy Av 31, 119991, Moscow, Russia
[4] Ecole Polytechnique Fédérale de Lausanne, EPFL-SB-ISIC-GPAO, Station 6, CH-1015, Lausanne, Switzerland
[5] N. N. Semenov Institute of Chemical Physics, Russian Academy of Sciences, Kosigin str. 4, 119991, Moscow, Russia

**SI1. Synthesis of Au-nanoparticles.**

Au-nanorods synthesized according to the protocol [1].
*Preparation of Au Seeds.* In a typical procedure, 50 μL of an aqueous 0.01 M solution of $HAuCl_4 \cdot 3H_2O$ was added to 2 mL of a 0.10 M CTAB solution. The solutions were gently stirred by shaking. Then, 24 μL of an aqueous 0.01M ice-cold $NaBH_4$ solution was added and mixed for 2 min. Care should be taken to allow the escape of the evolved gas during mixing. The solution developed a beige color. Then the solution was kept at 25°C for future use. This seed solution was used 2 h after its preparation.
*Preparation of Au Nanorods.* The 50 mL of 0.10 M CTAB, 2.5 mL of 0.01 M $HAuCl_4 \cdot 3H_2O$, and 0.4 mL of 0.01 M $AgNO_3$ solutions were added in that order consistently into the flask with stirring. Then 0.4 mL of 0.10 M ascorbic acid was added into the reaction mixture with stirring. The solution became colorless at this moment. Finally, 0.5 mL of seed solution was added, and the reaction mixture was gently mixed for 15 s and left undisturbed for at least 3 h. [Sau, T.K. and Murphy, C.J., 2004. Seeded high yield synthesis of short Au nanorods in aqueous solution. *Langmuir*, *20*(15), pp.6414-6420.]

The SEM image of particles is presented in Fig.2.

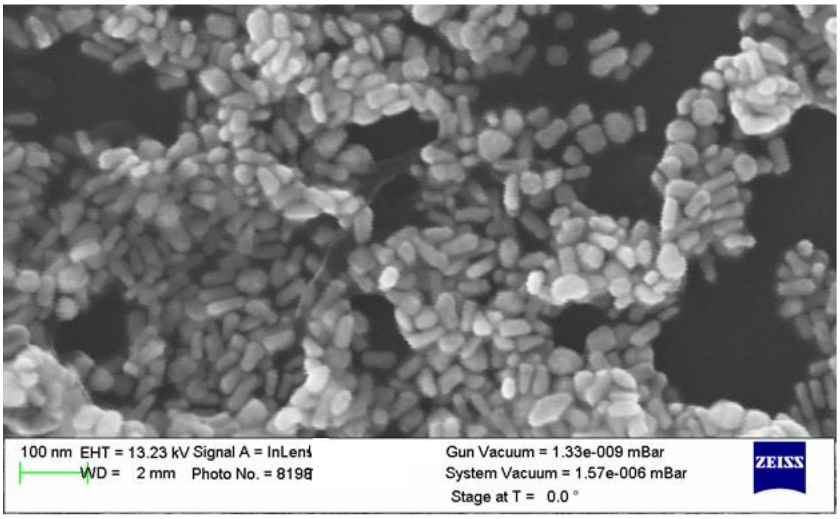


Fig. SI1. SEM image of AuNPs synthesized according to the protocol [1]

Gold Nanoparticle synthesized according to the protocol [2]. The standard method, as described by Turkevich [Turkevich, J., Stevenson, P.C. and Hillier, J., 1951. A study of the nucleation and growth processes in the synthesis of colloidal gold. *Discussions of the Faraday Society*, *11*, pp.55-75.], was used with some changes. The reduction of a hydrogen tetrachloroaurate(III) solution has been initiated by sodium tris-citrate by bringing gold solution to to a temperature of about the boiling. The reaction mixture is vigorously stirred by Teflon-coated magnetic bars. When the solution (147 µl 0.2M $HAuCl_4$ in water 100 ml) heats up to 95°C, the citrate solution (3 ml 0.034M) was added. After a 20 min the liquid was extracted and cooled to room temperature. The SEM image of particles is presented in Fig. SI1

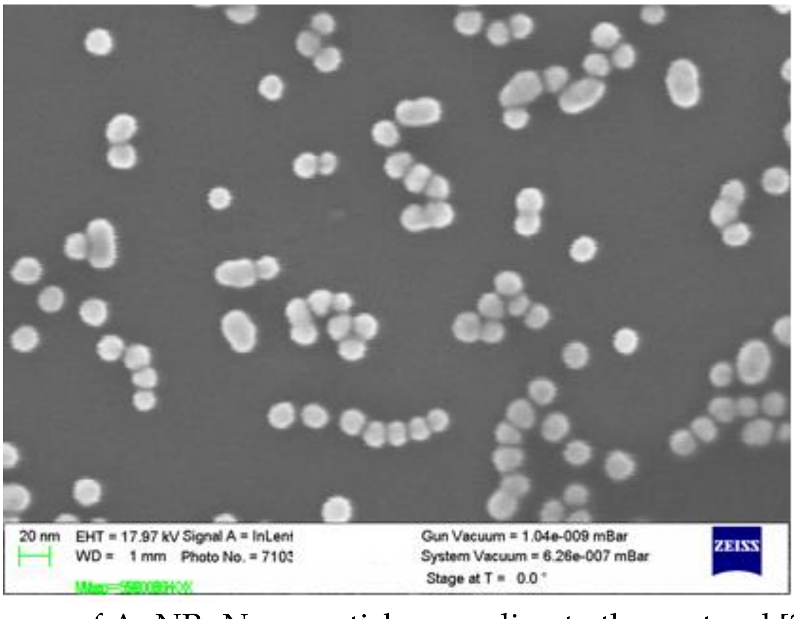


Fig. SI2. SEM image of AuNPs Nanoparticle according to the protocol [2].

1. T.K. Sau and C.J. Murphy. Langmuir 2004, 20, 6414-6420.
2. Turkevich, J.; Stevenson, P. C.; Hillier, J. Discuss. Faraday Soc. 1951, 11, 55.

## SI2. The spectrum of tungsten-halogen lamp.

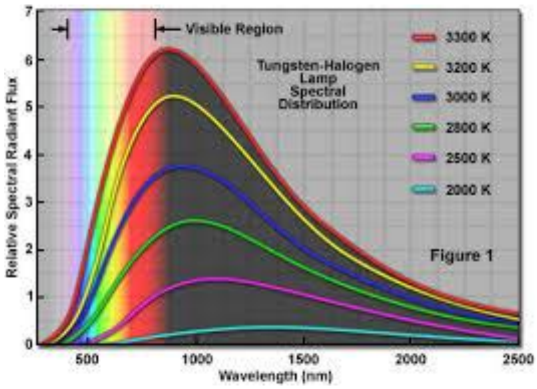


Figure SI3. The spectrum of tungsten-halogen lamp.

http://zeiss-campus.magnet.fsu.edu/articles/lightsources/tungstenhalogen.html

## SI3. Femtosecond laser photolysis setup.

Transient absorption spectra were measured by the femtosecond pump–supercontinuum probe setup.12 The pump was performed by the Gauss pulses with a repetition frequency of 15 Hz, time duration 25 fs, wavelength 740 nm, and energy 15 nJ. The white supercontinuum pulses generated in a quartz cell with H2O were used as probe pulses. The diameter of a probe spot was ~100 μm. The pump light spot has a diameter of 300 μm. The relative polarizations of the pump and probe beams were adjusted to 54.7° (magic angle) configurations. After the sample, the supercontinuum was dispersed by a polychromator («Acton SP-300») and detected by CCD camera («Roper Scientific SPEC-10»). Absorption difference spectra ΔA (t, λ) were recorded over the spectral range 400–740 nm. The measured spectra were corrected for group delay dispersion of the supercontinuum.

## SI4. The estimation of the local temperature at the surface of AuNPs in Au/TiO2 sample under steady state illumination.

The temperature of the sphere of the radius r in the continuous medium at the distance r from the center of the sphere T(r) is defined as (1)

$$T(r) = T_0 + \frac{W}{4\pi\lambda r} \quad (1)$$

The $T(r_0)$ at the sphere surface is (2)

$$T(r_0) = T_0 + \frac{W}{4\pi\lambda r_0} \tag{2}$$

Where W= $\sigma_{abs}$·I is the light power absorbing by sphere with the cross-section of the absorption $\sigma_{abs} = \pi r^2 Q_{abs}$. I is light intensity in W/m$^2$ units. $Q_{abs}$ is equal to 0.0019 for r=2.5 nm Au nanoparticles at the peak of the plasmon resonance. $Q_{abs}$ was calculated according to the Mi theory. The coefficient $\lambda = \chi/(c_p\,\varrho)$ is equal to 0,143 $10^{-6}$ m$^2$/s, where $\chi$ is heat transfer coefficient, $c_p$ is heat capacity and $\varrho$ is water density. The temperature increase for I= 1W/cm$^2$ is equal to 0.215 $^{o}$C according to equation (2).

**SI 5. Bright and dark field TEM images of Au/TiO2 NPs.**

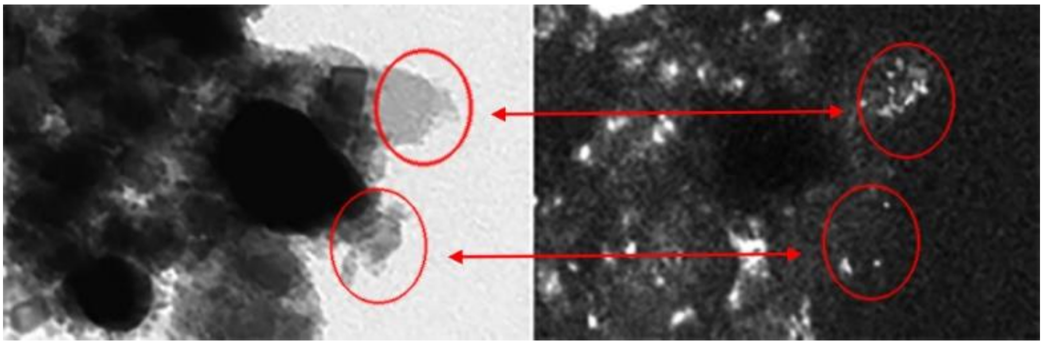

(a) (b)

Figure SI 4. Bright (a) and dark (b) field electron microscopy images of Au/$TiO_2$ NPs after 24h irradiation with LED green light (300 μW/cm$^2$). Red circles indicate fragments of NPs after irradiation. Bright spots in the dark field image suggest the presence of Au fragments.

**SI 6. The evaluation of bactericide effect of residual solution after the Au nanorods synthesis**.

The reaction mixture for the preparation of gold nanoparticles consisted of reagents: 0.10 M (36.4 g/l) CTAB, 0.47 mM $HAuCl_4$ (0.16 g/l), and 76 μM $AgNO_3$ (12.8 mg/l ) (Sau, T.K. and Murphy, C.J., 2004). $MIC_{CTAB}$ (*E. coli* AB1157) was determined to be equal to < 50 μg/ml. It is in reasonable agreement with previously reported data (Ishikawa, S. et al. 2002, Guo, L. et al. 2015). $MIC_{Ag+}$(*E. coli* AB1157) = 0.1 μg/ml (Radzig M.A. et al. 2013). $MIC_{Au3+}$ (*E. coli* AB1157) equals 1 μg/ml. The CTAB concentration after Au nanorods synthesis is conserved at the same level. Silver ions are catalyst and Ag+ concentration apparently is not changed significantly. The CTAB concentration exceeds $MIC_{CTAB}$ (*E. coli* AB1157) 7200 times, Ag+ concentration exceeds $MIC_{Ag+}$(*E. coli* AB1157) 128 times. These estimates show that the washing of the synthesized nanoparticles should be carried out very carefully.

Sau, T.K. and Murphy, C.J., 2004. Seeded high yield synthesis of short Au nanorods in aqueous solution. *Langmuir*, *20*(15), pp.6414-6420.

Ishikawa, S., Matsumura, Y., Yoshizako, F. and Tsuchido, T., 2002. Characterization of a cationic surfactant-resistant mutant isolated spontaneously from Escherichia coli. *Journal of applied microbiology*, *92*(2), pp.261-268.

Guo, L., Long, M., Huang, Y., Wu, G., Deng, W., Yang, X., Li, B., Meng, Y., Cheng, L., Fan, L. and Zhang, H., 2015. Antimicrobial and disinfectant resistance of E scherichia coli isolated from giant pandas. *Journal of applied microbiology*, *119*(1), pp.55-64.

Radzig, M.A., Nadtochenko, V.A., Koksharova, O.A., Kiwi, J., Lipasova, V.A. and Khmel, I.A., 2013. Antibacterial effects of silver nanoparticles on gram-negative bacteria: influence on the growth and biofilms formation, mechanisms of action. *Colloids and Surfaces B: Biointerfaces*, *102*, pp.300-306.

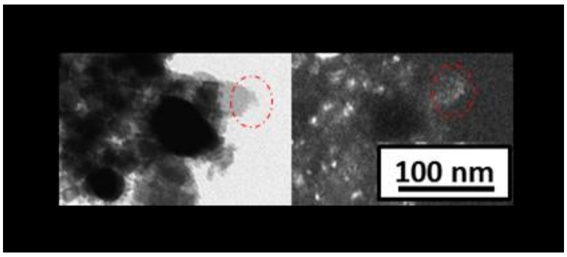

Supplement: Supplementary file 1 [file nanomaterials-09-00217-s001.pdf]
